# Facial Emotion Recognition in Children With Narcolepsy Type 1

**DOI:** 10.1111/jsr.70191

**Published:** 2025-08-26

**Authors:** Marco Veneruso, Paola Del Sette, Ramona Cordani, Antonella Barbieri, Lorenzo Chiarella, Serena Lecce, Cristina Venturino, Salvatore Lo Cascio, Francesco Biscarini, Fabio Pizza, Lino Nobili, Giuseppe Plazzi

**Affiliations:** ^1^ Dipartimento di Neuroscienze, Riabilitazione, Oftalmologia, Genetica e Scienze Materno‐Infantili Università di Genova Genova Italy; ^2^ UOC Neuropsichiatria Infantile, IRCCS Istituto Giannina Gaslini Genova Italy; ^3^ UO Psicologia Clinica e Psicoterapia, IRCCS Ospedale Policlinico San Martino Genova Italy; ^4^ Dipartimento di Scienze del Sistema Nervoso e Del Comportamento Università di Pavia Pavia Italy; ^5^ UOSD Psicologia, IRCCS Istituto Giannina Gaslini Genova Italy; ^6^ UO Neuropsichiatria Infantile, Promozione Della Salute, Materno‐Infantile, di Medicina Interna e Specialistica di Eccellenza “G. D'Alessandro” Palermo Italy; ^7^ Dipartimento di Scienze Biomediche e Neuromotorie, Alma Mater Studiorum Università di Bologna Bologna Italy; ^8^ IRCCS Istituto Delle Scienze Neurologiche di Bologna Bologna Italy; ^9^ Università di Modena e Reggio Emilia, Dipartimento di Scienze Biomediche, Metaboliche e Neuroscienze Modena Italy

**Keywords:** emotion recognition, narcolepsy, neurodevelopment, social cognition, social functioning, theory of mind

## Abstract

Narcolepsy type 1 is a neurological disorder typically emerging in childhood or adolescence, characterised by excessive daytime sleepiness, cataplexy and rapid eye movement sleep‐related symptoms. Beyond its core features, increasing evidence suggests an impact on socio‐cognitive development, including difficulties in understanding others' mental states. In this study, we aimed to clarify whether such impairments extend to more basic emotional processes. We expanded previous work on Theory of Mind by including an analysis of facial emotion recognition and comparing children with narcolepsy type 1 to a newly recruited group of typically developing peers. Twenty‐two children with narcolepsy type 1 and twenty‐two age‐ and sex‐matched controls completed standardised tasks assessing Theory of Mind and facial emotion recognition. Results confirmed a significant impairment in Theory of Mind among children with narcolepsy compared to controls. In contrast, no differences emerged in facial emotion recognition, suggesting a selective disruption in higher‐order socio‐cognitive processes. Additionally, greater daytime sleepiness was associated with poorer Theory of Mind performance, but not with emotion recognition accuracy. These results indicate a specific vulnerability in social understanding during the development of type 1 narcolepsy, probably aggravated by narcoleptic symptoms. However, facial emotion recognition is not affected in these patients, suggesting the involvement of different networks. Early identification of these difficulties and targeted interventions may be crucial to support peer relationships and long‐term psychosocial outcomes in affected children.

## 1 Introduction

Narcolepsy type 1 (NT1) is a central nervous system disorder characterised by excessive daytime sleepiness (EDS), cataplexy (a sudden loss of muscle tone triggered by emotions), and various REM sleep‐related disturbances, such as sleep paralysis, hallucinations, disrupted nighttime sleep and REM sleep behaviour disorder (Plazzi et al. 2018). This condition is linked to a deficiency in hypocretin‐1, a neurotransmitter essential for regulating wakefulness, likely due to an autoimmune process against the hypocretin‐producing neurons (Latorre et al. 2018).

NT1 often emerges in childhood or adolescence, but the symptoms in children can differ notably from those in adults, complicating timely recognition and diagnosis. For instance, children with NT1 may display behavioural changes linked to sleepiness and can experience cataplexy without recognisable emotional triggers (Plazzi et al. 2018). These distinct presentations suggest a deep understanding of how NT1 affects the social and emotional development of young patients (Thorpy and Krieger 2014).

The emotional component of NT1 is particularly relevant, as emotional stimuli—positive or negative—can provoke cataplexy episodes (Krahn et al. 2005). Studies indicate that NT1 patients exhibit structural and functional changes in the brain's limbic areas, which are central to emotional processing (Brabec et al. 2011; Ponz et al. 2010a, 2010b; Schiappa et al. 2018). However, research on the behavioural impact of these alterations shows not univocal results. In adults, a study on NT1 patients found no significant differences in their ability to recognise facial emotions compared to healthy controls. This may suggest compensatory mechanisms that support emotion recognition in adulthood (Bayard et al. 2013). Conversely, our study on children with NT1 highlighted a selective impairment in Theory of Mind (ToM) (Veneruso et al. 2024). ToM is the ability to understand others' mental states (i.e., intentions, desires, beliefs and emotions) to interpret, predict, and manipulate social behaviour (Premack and Woodruff 1978). This socio‐cognitive skill involves a higher‐level cognitive aspect than simply recognising facial emotions (Turner and Felisberti 2017). Indeed, its development started in early childhood and continued throughout the lifespan (Del Sette et al. 2023). Finding a ToM impairment in children with NT1, we suggested a specific socio‐cognitive difficulty in youths that could be detected through tasks sensitive to individual differences like ToM tasks.

The contrasting results may be due to age‐related differences: children with NT1 might lack the alternative compensatory networks that adults develop over time, making them more vulnerable to difficulties in interpreting complex social cues. Another possibility is that socio‐cognitive challenges in NT1 are specific to tasks that require inferring others' mental states in complex social situations, rather than basic emotion recognition.

In this study we aim to understand the impact of NT1 on facial emotion recognition in children, confirming the presence of a selective deficit in ToM skills as shown in our previous study.

## 2 Methods

### 2.1 Participants

We recruited children aged 8–13 years with a diagnosis of NT1, according to the International Classification of Sleep Disorders Third Edition (American Academy of Sleep Medicine 2023), from the outpatient clinic at the child neuropsychiatry unit of the Gaslini Institute in Genoa and the narcolepsy center at the Institute of Neurological Sciences in Bologna. The sample included the same participants described in our previous study (Veneruso et al. 2024). Although emotion recognition data were collected at that time, they were not analysed or published. In the present study, we compared these existing NT1 data with a newly recruited control group, assessed with the same tasks, to both replicate our previous ToM findings and newly investigate emotion recognition abilities.

Indeed, a new control group was formed by consecutively recruiting children attending the outpatient clinic at the orthopaedics unit of the Gaslini Institute in Genoa, matched for age and sex. All children had good proficiency in the Italian language and no history of developmental delay.

Written informed consent to participate in the study was obtained from all subjects and their parents. The study was approved by the Liguria Regional Ethics Committee (453/2022‐DB id 12567).

NT1 patients and controls underwent a series of tests and questionnaires designed to assess ToM abilities, emotion recognition, and sleepiness. Evaluations were conducted in the afternoon in an artificially lit room, following a 15‐ to 20‐min nap to minimise the potential impact of sleepiness on performance.

### 2.2 Emotion Recognition

Children's ability to recognise others' emotions from facial expressions was assessed using the child version of the Reading the Mind in the Eyes Test (RMET) (Baron‐Cohen et al. 2001). In this task, children were shown 28 photographs of different individuals' faces, each accompanied by four words or phrases describing possible emotions. The children were asked to select the word or phrase that best described each individual's emotional expression. The total Emotion Recognition score, ranging from 0 to 28, was calculated based on the number of correct responses and was used as a variable of interest in the analyses.

### 2.3 Theory of Mind

Theory of Mind was assessed using the Strange Stories (SS) task (Happé 1994) and the Silent Films task (Devine and Hughes 2016).

The SS task evaluates children's ability to understand others' mental states within complex social scenarios presented in written format. Five mental stories were administered: two involving double bluffs, two involving misunderstandings and one involving persuasion. After each story, children were asked to explain, in writing, the reasoning behind the main character's behaviour, requiring them to make inferences about the character's mental states in context. Scoring followed established guidelines (White et al. 2009): 0 points were given for incorrect or ‘don't know’ answers, 1 point for partially correct and implicit answers, and 2 points for fully correct and explicit answers. The total SS mental score was calculated by summing the scores from each story, yielding a possible range from 0 to 10.

The SF task assesses children's ability to interpret others' mental states based on observed behaviour. Five short clips (average length = 25.4 s) from Harold Lloyd's classic silent comedy *Safety Last* were shown. After viewing each clip, children were required to explain the main character's behaviour. Following the scoring criteria, 0 points were given for incorrect or ‘don't know’ answers, 1 point for partially correct responses, and 2 points for fully accurate and explicit responses. The total SF score was obtained by summing the scores from each clip, with a possible range from 0 to 10.

### 2.4 Clinical Evaluation

Children diagnosed with NT1 underwent a comprehensive clinical evaluation, which included an interview aimed at assessing the severity of symptoms such as daytime sleepiness, cataplexy, sleep paralysis, and hypnagogic hallucinations. Additional information was collected on the age at narcolepsy onset, the time delay between onset and diagnosis, and the use of medications or behavioural therapies.

To quantify subjective excessive daytime sleepiness (EDS), the Epworth Sleepiness Scale for Children and Adolescents (ESS‐CHAD) (Janssen et al. 2017) was administered to all participants. This questionnaire comprises eight items, each rated from 0 (‘never’) to 3 (‘always’), with each item depicting a situation in which the patient evaluates the likelihood of falling asleep. Healthy controls were also assessed for sleepiness using the ESS‐CHAD.

### 2.5 Statistical Analysis

Data were examined for normal distribution by performing the Shapiro‐Wilk Test. Since some variables were not normally distributed and due to the small sample size, we decided to use nonparametric tests.

Group differences were analysed by performing the Mann‐Whitney U test. Relationships between variables were tested through non‐parametric correlations (i.e., Spearman's Rho) and regression analyses.

Statistical analyses were performed with SPSS version 28.0 for Windows (SPSS, Chicago, USA).

The level of significance was *α* < 0.05.

## 3 Results

This study included 22 children with NT1 (six female; age: Q1 = 9.10, median = 11.4, Q3 = 12.1, range: 8.0–13.5) and 22 typically developing children (six female; age: Q1 = 9.9, median = 11.7, Q3 = 12.67, range: 7.9–13.1). The two groups were matched for age (*U* = 263.0, *p* = 0.630) and sex.

For the NT1 group, the median age at disease onset was 7.95 years, with a median diagnostic delay of 0.65 years (SD^2^ = 0.85) and a median disease duration of 2.81 years. All NT1 patients had CSF hcrt‐1 levels below 110 pg/mL, and 21 (99.46%) were positive for HLA‐DQB1*0602. In terms of treatment, five patients (23%) were treatment‐naive, whereas 17 (77%) were receiving drug therapy, and nine (40.9%) were also engaged in behavioural therapy. Regarding symptom severity, the ESS‐CHAD median score was 13, and the median cataplexy frequency score was 4. Within the NT1 group, two patients reported one or fewer cataplectic attacks per year, two reported one or more attacks per month but fewer than one per week, nine experienced one or more attacks per week but fewer than one per day, and nine reported at least one cataplectic attack per day.

### 3.1 Group Differences

Table 1 shows descriptive statistics. Children with NT1 reported higher sleepiness (*U* = 0.00, *p* < 0.001) and scored significantly lower than healthy controls on ToM tasks, as indicated by the SF (*U* = 139.5, *p* = 0.015) and SS (*U* = 153.0, *p* = 0.034). In contrast, no significant differences emerged between groups for facial emotion recognition (*U* = 215.0, *p* = 0.523).

**TABLE 1:** Descriptive statistics and group differences (Control vs. Clinical group).

	Control group *N* = 22			NT1 group *N* = 22			Group differences	
	Median	Q1–Q3	Range	Median	Q1–Q3	Range	Mann–Whitney U	*p*
SS	8.00	6.75–8.00	4.00–10.00	5.00	4.00–8.00	2.00–9.00	153.00	0.034
SF	7.50	6.00–10.00	2.00–11.00	6.00	3.75–7.00	2.00–10.00	139.50	0.015
RMET	19.00	16.00–20.00	15.00–24.00	18.00	16.00–21.00	14.00–23.00	215.00	0.523
ESS CHAD	3.50	1.75–5.00	0.00–6.00	13.00	10.75–15.25	7.00–22.00	0.00	< 0.001

Abbreviations: ESS CHAD, Epworth Sleepiness Scale Children and Adolescents; RMET, Reading the Mind in the Eyes Test; SF, Silent Film; SS, Strange Stories.

### 3.2 Correlations

Sleepiness correlated negatively with ToM, but not RMET (Table 2). Interestingly, this correlation exists only in the NT1 group and not in the control group. Regression analyses with ToM as dependent variables and sleepiness, group, and their interaction as independent variables revealed that SS and SF scores were predicted only by EDS score, *R* ^2^ = 0.31, *β* = −0.55; *p* < 0.001, *R* ^2^ = 0.23, *β* = −0.48; *p* = 0.001, respectively.

**TABLE 2:** Spearman's correlations between socio‐cognitive skills and daytime sleepiness.

	Total *N* = 44			Control group *N* = 22			NT1 group *N* = 22		
	SS	SF	RMET	SS	SF	RMET	SS	SF	RMET
ESS CHAD	−0.45**	−0.48**	−0.04	−0.25	−0.09	0.15	−0.48*	−0.45*	0.10

*Note*: **p < 0.01. *p < 0.05.

Abbreviations: ESS CHAD, Epworth Sleepiness Scale Children and Adolescents; RMET, Reading the Mind in the Eyes Test; SF, Silent Film; SS, Strange Stories.

## 4 Discussion

This study aimed to expand the understanding of socio‐cognitive functioning in children with NT1 by evaluating both ToM abilities and facial emotion recognition. Building upon previous findings, which identified significant ToM impairments in this population, we sought to determine whether these difficulties extend to more fundamental emotional processing skills, namely emotion recognition. Although ToM data from the NT1 group were previously reported (Veneruso et al. 2024), emotion recognition was assessed during the same testing session using the RMET, but those data were neither analysed nor published at the time. In the present study, we newly analysed these RMET data and introduced a novel control group assessed with both ToM and RMET tasks, allowing for a direct comparison across socio‐cognitive domains.

Although the results on ToM skills were confirmed, children with NT1 showed a preserved ability to recognise basic facial emotions, comparable to that of typically developing peers. These findings provide further insights into how NT1 may selectively impact peculiar aspects of social cognition during childhood.

Indeed, this pattern suggests that socio‐cognitive impairments in NT1 are not generalised but instead specifically affect higher‐order cognitive processes involved in understanding others' mental states. Theory of Mind requires complex inferential reasoning, perspective‐taking, and the integration of social cues—all of which rely on the coordinated activity of multiple brain regions, including the medial prefrontal cortex, temporoparietal junction, posterior middle temporal gyrus and the inferior frontal gyrus (Byom and Mutlu 2013; Del Sette et al. 2023). In contrast, facial emotion recognition primarily engages more automatic and perceptual processes that involve similar but not identical brain networks (Byom and Mutlu 2013). This fits with the previous hypothesis that neural alterations involving emotional processing in NT1 manifest in an alteration of ToM but not emotion recognition (Del Sette et al. 2023).

One possible explanation for the selective impairment in ToM could be the impact of EDS, a core symptom of NT1 (Overeem et al. 2001).

The negative correlation between EDS and ToM performance further supports the idea that sleepiness exacerbates difficulties in complex cognitive tasks. Studies on adolescents showed a negative impact of EDS on executive functions, attention, and working memory, skills that are essential for understanding others' mental states (Anderson et al. 2009; Sun et al. 2022; Wade et al. 2018). Interestingly, in line with previous studies, we did not observe a significant relation between EDS and emotion recognition, suggesting that recognising basic facial emotions may rely on more automatic, less cognitively demanding processes that remain unaffected by sleepiness (Van Der Helm et al. 2010). This distinction highlights the need to consider how NT1 symptoms, particularly sleepiness, differentially impact specific aspects of socio‐cognitive functioning.

Our findings fit within the developmental literature showing that ToM develops during early and middle childhood, within the context of social relationships, and plays a crucial role in social relationships during childhood (Caputi et al. 2012; Carpendale and Lewis 2004; Lecce et al. 2017). For these reasons, ToM disruption, during developmental stages, as could happen due to NT1 symptoms, could have long‐lasting effects on social functioning. These aspects may explain potential differences between patients with childhood‐onset NT1 and those with adult‐onset NT1. Unfortunately, to our knowledge, this socio‐cognitive domain has never been investigated in adults, and no longitudinal studies have tracked how these abilities develop in children with NT1 as they grow older.

From a clinical point of view, these results underscore the importance of comprehensive socio‐cognitive assessments in children with NT1. Deficits in ToM can significantly impact social interactions and peer relationships, possibly leading to social isolation (Posar et al. 2020; Prihodova et al. 2018; Veneruso et al. 2024). Children who struggle to interpret others' thoughts, intentions and emotions may be at greater risk for peer rejection and developing social anxiety symptoms (Ronchi et al. 2020). Early identification of these challenges is critical for implementing targeted interventions, already proved to be effective during early and middle childhood in typically developing children, aimed at improving social cognition (Bianco et al. 2016; Fisher and Happé 2005; Lecce, Bianco, Demicheli, and Cavallini 2014; Lecce, Bianco, Devine, et al. 2014; Paynter and Peterson 2013).

Future research should explore the effect of puberty on the socio‐cognitive differences in NT1 children. Indeed, puberty may begin earlier in children with NT1 and is a critical developmental period, marked by significant neurobiological changes, also in brain regions involved in social cognition (Burnett et al. 2011; Poli et al. 2013). Although some evidence suggests that puberty can influence facial social cognition (e.g., (Lawrence et al. 2015)) other findings indicate that puberty may not have a direct effect (Vetter et al. 2018). Studies that explore this with stratified analyses based on pubertal status may help clarify the complex interplay between neurodevelopmental trajectories and social‐cognitive functioning in both clinical and typically developing populations.

Additionally, effective management of EDS through pharmacological and behavioural strategies may indirectly support social functioning by improving attention and reducing cognitive fatigue (Chin et al. 2022).

Limitations must be acknowledged and future studies with larger and more diverse samples are needed to validate these results. The relatively small sample size and the cross‐sectional design of this study preclude conclusions about causality or developmental relationships.

Longitudinal and training research is essential to examine how socio‐cognitive abilities develop in NT1 and to determine whether interventions targeting sleepiness and cognitive functioning can mitigate ToM deficits.

Another limitation concerns the socio‐cognitive tasks used. Future research would benefit from examining how children with NT1 interpret and react to dynamic social scenarios, as real‐life interactions typically involve more complex emotional cues than those presented in standardised assessments.

Finally, we acknowledge the statistical limitations imposed by the relatively small sample size. Although this study aimed to explore novel aspects of socio‐cognitive functioning in NT1, the sample size restricted our ability to include multiple control variables in the regression models. To minimise the risk of overfitting, we adopted a parsimonious analytic strategy, including only the most theoretically relevant factor (trait sleepiness). We did not include working memory or other cognitive measures as covariates, as previous findings in this population did not reveal significant group differences in this domain. Nonetheless, future studies with larger samples will be essential to disentangle the relative contributions of sleepiness, cognitive load and neurodevelopmental factors to the socio‐cognitive phenotype of NT1.

Future studies should also investigate the impact of other factors, such as medication use, disease severity, and comorbid conditions (e.g., ADHD, anxiety) on socio‐cognitive functioning. Finally, exploring how emotional regulation and coping strategies influence social functioning could offer valuable insights, particularly given the role of emotional triggers in cataplexy.

In conclusion, the present study confirmed the presence of ToM impairments in children with Narcolepsy Type 1 and extended previous findings (Veneruso et al. 2024) showing that this deficit was specific to ToM while facial emotion recognition remains intact. This selective deficit highlights the need for comprehensive assessments of socio‐cognitive functioning in NT1 and supports the development of targeted interventions. Early detection and personalised interventions focusing on social cognition could significantly improve social relationships and overall quality of life in this vulnerable population.

## Author Contributions

**Marco Veneruso:** conceptualization, investigation, writing – original draft, methodology, writing – review and editing, formal analysis, data curation. **Paola Del Sette:** conceptualization, writing – review and editing, writing – original draft, methodology. **Ramona Cordani:** writing – review and editing, conceptualization, methodology. **Antonella Barbieri:** writing – review and editing, investigation. **Lorenzo Chiarella:** writing – review and editing, investigation. **Serena Lecce:** conceptualization, methodology, writing – review and editing. **Cristina Venturino:** writing – review and editing. **Salvatore Lo Cascio:** writing – review and editing. **Francesco Biscarini:** writing – review and editing. **Fabio Pizza:** writing – review and editing, methodology. **Lino Nobili:** conceptualization, methodology, writing – review and editing, supervision. **Giuseppe Plazzi:** conceptualization, methodology, writing – review and editing, supervision.

## Conflicts of Interest

The authors declare no conflicts of interest.

## Data Availability

The data that support the findings of this study are available on request from the corresponding author. The data are not publicly available due to privacy or ethical restrictions.
